# Autophagy attenuates high glucose-induced oxidative injury to lens epithelial cells

**DOI:** 10.1042/BSR20193006

**Published:** 2020-03-31

**Authors:** Xiaomin Liu, Xiaowen Zhao, Rong Cheng, Yusen Huang

**Affiliations:** 1 Qingdao University Medical College, Qingdao, China; 2 State Key Laboratory Cultivation Base, Shandong Provincial Key Laboratory of Ophthalmology, Shandong Eye Institute, Shandong First Medical University and Shandong Academy of Medical Sciences, Qingdao, China

**Keywords:** autophagy, diabetic cataract, high glucose, lens, oxidative stress

## Abstract

**Purpose:** Autophagic dysfunction and abnormal oxidative stress are associated with cataract. The purpose of the present study was to investigate the changes of cellular autophagy and oxidative stress and their association in lens epithelial cells (LECs) upon exposure to high glucose.

**Methods:** Autophagy and oxidative stress-related changes were detected in streptozotocin-induced Type 1 diabetic mice and normal mouse LECs incubated in high glucose conditions. Rapamycin at a concentration of 100 nm/l or 50 μM chloroquine was combined for analysis of the relationship between autophagy and oxidative stress. The morphology of LECs during autophagy was observed by transmission electron microscopy. The expressions of autophagy markers (LC3B and p62) were identified, as well as the key factors of oxidative stress (SOD2 and CAT) and mitochondrial reactive oxygen species (ROS) generation.

**Results:** Transmission electron microscopy indicated an altered autophagy activity in diabetic mouse lens tissues with larger autophagosomes and multiple mitochondria. Regarding the expressions, LC3B was elevated, p62 was decreased first and then increased, and SOD2 and CAT were increased before a decrease during 4 months of follow-up in diabetic mice and 72 h of culture under high glucose for mouse LECs. Furthermore, rapamycin promoted the expressions of autophagy markers but alleviated those of oxidative stress markers, whereas chloroquine antagonized autophagy but enhanced oxidative stress by elevating ROS generation in LECs exposed to high glucose.

**Conclusions:** The changes in autophagy and oxidative stress were fluctuating in the mouse LECs under constant high glucose conditions. Autophagy might attenuate high glucose-induced oxidative injury to LECs.

## Introduction

Cataract occurs two to five times more frequently in patients with diabetes than nondiabetic populations [1,2]. Diabetic cataract (DC), one of the major ocular complications of hyperglycemia, often develops in earlier age of diabetic patients and progresses fast [3,4]. Moreover, approximately a quarter of patients with late-onset diabetes will undergo cataract surgery within 10 years [2]. Lens epithelial cells (LECs), as the major tissue participating in nutrition and ions transportation, metabolism, and detoxification during lens development [5], contribute greatly to transparency of the lens [6]. Exposure of LECs to high glucose (HG) may be one of the reasons for DC formation, but the detailed pathogenesis remains largely unknown.

Autophagy is an evolutionally conserved catabolic process, which involves the degradation of cytosolic macromolecules and membrane-bound organelles through lysosome machinery and maintains the balance among the synthesis, degradation, and subsequent recycling of cellular components [7]. Autophagosomal–lysosomal pathway is critically important for maintaining lens transparency in clearing the degradation of LEC proteins and organelles. Our group previously elucidated a high level of autophagy activity in LECs under HG conditions [8]. However, overactivation of autophagy may result in cell death [9], and it is difficult to maintain the intracellular homeostasis for insufficient autophagy. Malfunction of autophagy has been linked to many types of cataracts, such as hereditary cataract [10,11], congenital cataract [12], age-related cataract [13], and DC [8]. Meanwhile, the molecular mechanism changes of autophagy activity during the development of DC are weakly understood. Further investigations are necessary on the effect of autophagy in the pathogenesis of DC.

Hyperglycemia-induced oxidative stress is a major risk factor of diabetes-associated complications. Several mechanisms related to hyperglycemia have been identified, such as increased levels of mitochondrial reactive oxygen species (ROS) in the affected tissues [14]. ROS are thought to induce autophagy, while autophagic dysfunction in turn results in ROS accumulation [15,16]. The excessive production or imbalance of ROS can cause oxidative damage to lens fibers [17], which contributes to the protein aggregation, light scatter, and cataract formation. In addition, the accumulation of ROS was observed to dysregulate the expressions of superoxide dismutase (SOD) and catalase (CAT) in HG conditions. SOD1 and CAT were increased in human gingival fibroblasts after 72 h of exposure to 25 mM glucose [18]. SOD2 and CAT were decreased in HK-2 cells under 30 mM HG ambience for 24 h [19]. Raju et al. [20] stated SOD2 expression was elevated within 24 h and reduced after 72 h in LECs upon exposure to 25 mM HG *in vitro*. Liu et al. [21] demonstrated SOD2 was higher in the first 4 weeks followed by a downstream trend for 8 weeks in the corneas of diabetic mice. However, the changes of oxidative stress in the LECs *in vivo* under HG conditions remain unclear.

Moreover, low glucose-induced oxidative stress could trigger autophagy in LECs. [22] During the aging process, cellular redox balance was broken down, [23] and autophagy activities attempted to restore lens homeostasis, but their failure may produce more ROS and oxidation [24]. Since the association among HG, autophagy, and oxidative stress in LECs seems to be elusory, streptozotocin-induced Type 1 diabetic mice and HG-cultured LECs from the capsular bag were used in the present study to investigate the changes in cellular autophagy and oxidative stress as well as their specific correlations.

## Materials and methods

The present study was approved by the Institutional Animal Care and Use Committee of Shandong Eye Institute. All procedures and animal handling were carried out in accordance with the Association for Research in Vision and Ophthalmology (ARVO) Statement for the Use of Animals in Ophthalmic and Vision Research. The experiments with animals were all performed at the labs of Shandong Eye Institute.

### *In vivo* experimental procedures

C57BL/6 male mice, 6 to 8 weeks old, were purchased from the Institute of Laboratory Animal Sciences, Chinese Academy of Medical Sciences (Beijing, China). Type 1 diabetes mellitus was induced in 60 mice by intraperitoneal injections of low-dose streptozotocin (50 mg/kg; Sigma-Aldrich, St. Louis, MO, U.S.A.) for 5 consecutive days. Sixty control mice were injected with 0.01 M citrate buffer solution. Animals with a blood glucose level higher than 16.7 mmol/l at 12 weeks after streptozotocin injection were considered to have diabetes [25] and used at 1, 2, 3, and 4 months, respectively, following the successful establishment of the mouse model. The mice were killed with pentobarbitone (100 mg/kg). The features of diabetic mice are presented in Table 1.

**Table 1:** Characteristics of the diabetic mice

Duration after the establishment of the diabetes model (months)	1	2	3	4
Blood glucose (mmol/l)	25.37 ± 2.06	24.40 ± 5.41	26.67 ± 2.40	25.77 ± 2.80
Weight (g)	20.90 ± 0.14	19.69 ± 0.47	18.94 ± 0.31	18.77 ± 0.28

### *In vitro* mouse capsular bag culture

Lenses were removed from mouse eyes using forceps. After submersion in DMEM/F12 supplemented with 10% fetal bovine serum (FBS) (Sigma-Aldrich, St. Louis, MO, U.S.A.), the lens was placed in a 3-cm dish with the anterior capsule facing down and the posterior capsule facing up. After the posterior capsule was excised along the lens equator, and the lens cortex was removed, the anterior capsule containing LECs was flattened on the bottom of the culture dish, allowing the growth of cells outward from the edge of the capsule. After 24 h, the LECs attaching to the dish bottom were treated with 5 mM glucose (normal glucose group, NG group) and 30 mM glucose (HG group) for another 24, 48, and 72 h, respectively. Moreover, the cells in the HG group were exposed to rapamycin at a concentration of 100 nm/l [26] (HG+RAPA group) and 50 μM chloroquine [27] (HG+CQ group) during the last 12 h of HG treatment, respectively.

### Transmission electron microscopy

Both normal and diabetic mouse lens tissues were fixed with 2.5% glutaraldehyde for 4 h or longer and then with 1% osmic acid for 1 to 1.5 h. The lenses were dehydrated in 50%, 70%, 90%, and 100% acetone three times, respectively, each for 15 min, before embedded in epoxy resin (EMS, Epon 812, 14120). Sections, 70 nm in thickness, were cut (Reichert-Jung ULTRACUT) and collected with a copper net. After stained with uranyl acetate and lead citrate, each for 15 min, the sections were viewed under a transmission electron microscope (JEM1200; Jeol, Tokyo, Japan).

### Immunohistochemistry

Eye globes from diabetic and control mice were fixed in 4% paraformaldehyde overnight and processed for paraffin embedding. Antigen retrieval was obtained by heating tissue slides in 0.01 M citrate buffer, pH 6.0, at 95°C for 20 min, after which endogenous peroxidate activity was blocked by 0.6% hydrogen peroxide for 10 min. Nonspecific staining was blocked by 5% normal goat serum for 1 h. Sections were then incubated with anti-LC3B, anti-p62, anti-SOD2, and anti-CAT rabbit primary antibodies (Supplementary Table S1) overnight at 4°C. After three washes with PBS, they were incubated with biotinylated rabbit anti-goat IgG (1:100) for 1 h, followed by DAB staining for 2 min. Rabbit primary antibody isotype controls were used as negative controls. The StrataQuest software was employed to quantify the relative levels of protein expressions.

### Quantitative real-time PCR

Total RNA was isolated using the GeneJET RNA Purification Kit (Thermo Fisher Scientific, Waltham, MA, U.S.A.) and quantified by spectrophotometry. cDNA was synthesized from 500 ng of RNA with oligo (dT) primers by using the cDNA Synthesis Kit (Takara, Dalian, China) and measured by quantitative real-time PCR (qRT-PCR; Applied Biosystems, Foster City, MA, U.S.A.). The primers used are shown in Supplementary Table S2.

### Western blotting analysis

Total protein was extracted from lysed samples (20 µg) of the diabetic mouse lens capsule or the LECs of the cultured capsular bag under different treatment conditions and run on 10–15% SDS-PAGE gels before transferred to a PVDF membrane (Millipore, Billerica, MA, U.S.A.). The samples were blocked with 5% nonfat dry milk and incubated with primary antibodies (Supplementary Table S1) overnight at 4°C. The blots were washed three times and incubated with a HRP-conjugated secondary antibody (Amersham Biosciences, Piscataway, NJ, U.S.A.). Finally, the blots were visualized via enzyme-linked chemiluminescence using the ECL kit (Pierce Biotechnology, Rockford, IL, U.S.A.) and quantified by using ImageJ software.

### Measurement of mitochondrial ROS generation

After treated with different concentrations of glucose and rapamycin or chloroquine, the cultured capsular bag tissues were removed to retain the LECs, which were preloaded with 50 nM Mitotracker green (Beyotime, Shanghai, China) for 20 min and 2.5 μM MitoSOX™ red reagent (Invitrogen, Carlsbad, CA, U.S.A.) for 10 min. The staining was observed and captured using a confocal microscope (TE2000-U, Nikon, Tokyo, Japan). The vigorous and uniform LECs of four quadrants were selected for photography and analysis. In each group, if any three of four images were observed to be obviously varied, significant difference was confirmed.

### Statistical analysis

All data were obtained from at least three repeated experiments and presented as means ± standard deviation (SD). SPSS 17.0 software (SPSS, Chicago, IL, U.S.A.) was used for statistical analyses with the Student’s *t*-test, and a *P* value of < 0.05 was considered statistically significant.

## Results

### HG-induced autophagy activity of LECs

**Figure 1:**
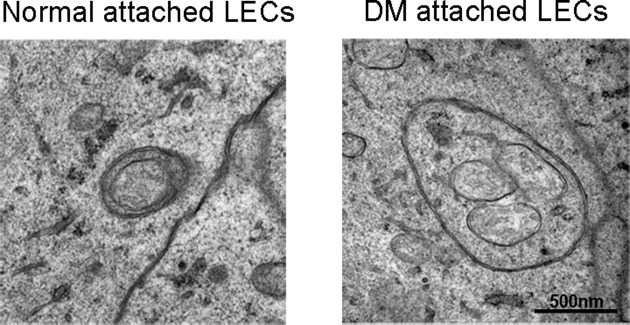
Electron micrographs of double membrane autophagosomes in lens epithelial cells (LECs) The autophagosomes, larger than the normal, encapsulated several mitochondria in LECs of a diabetic mellitus mouse.

Transmission electron microscopy showed double-membrane autophagosomes in both the normal and diabetic mouse lenses (Figure 1). However, the autophagosomes in the LECs of diabetic mice were larger than those in the normal. Moreover, multiple mitochondria were encapsulated in an autophagosome in diabetic LECs, indicating an altered autophagy activity induced by hyperglycemia.

### Alteration of autophagic response and oxidative stress in diabetic mice

**Figure 2:**
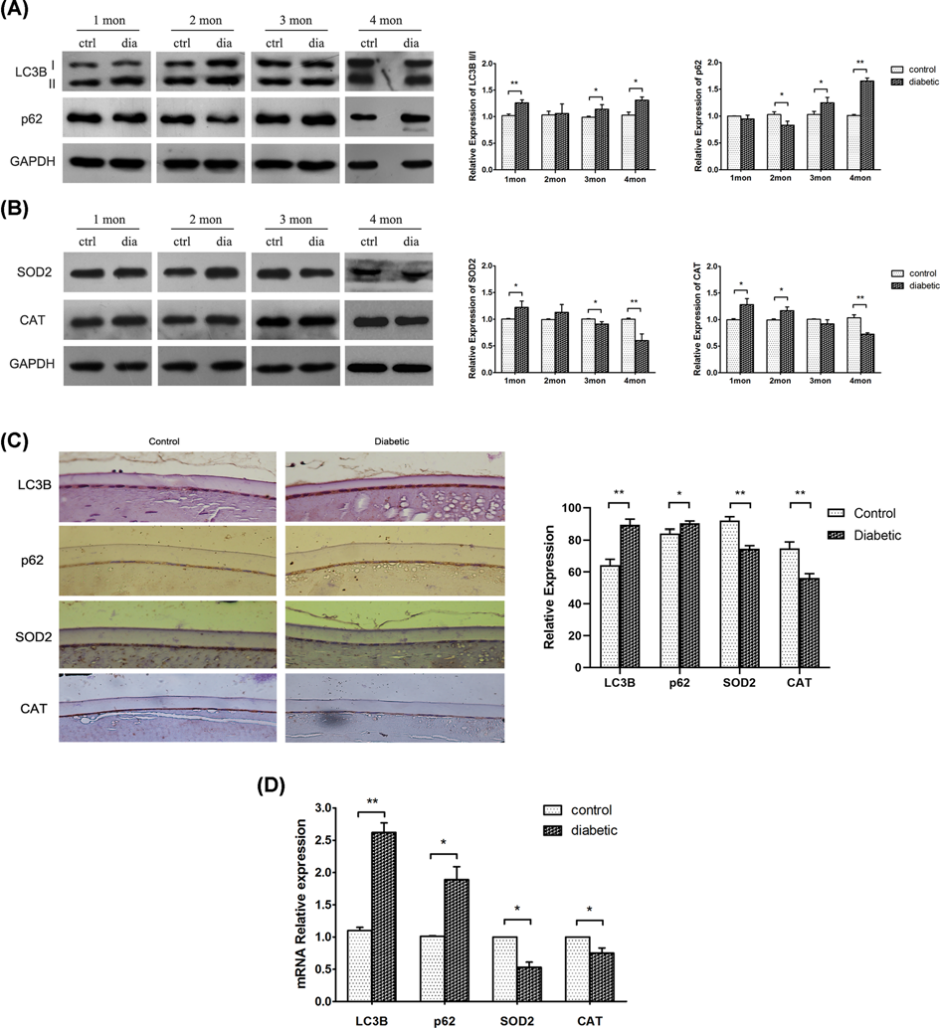
The expressions of autophagy and oxidative stress substrates in LECs of diabetic mice The diabetic mice were killed at 1, 2, 3, and 4 months after being confirmed as model for detection of autophagy (**A**) and oxidative stress (**B**) (ctrl = control, dia = diabetic). The LC3B II/I ratio was elevated during the follow-up, but p62 was reduced in the first 2 months and then increased. SOD2 and CAT were increased in the first 2 months, followed by an obvious reduction. (**C**) Immunohistochemistry showed the markers of autophagy were increased, and the factors of oxidative stress were reduced in LECs of diabetic mice at 4 months. (**D**) qRT-PCR showed the two markers of autophagy were increased, and the two factors of oxidative stress were decreased in LECs of diabetic mice at 4 months. *P<0.05 and **P<0.01 versus the control group (Student’s *t*-test). Data are mean ± SD (A and B, *n*=3; D, *n*=9).

Markers of the reactions in mice with a long time of hyperglycemia were identified. As shown in Figure 2A, the LC3B II/I ratio was elevated during the observation period of 4 months, although there was no significant difference at 2 months after the diabetes model was successfully established. Inversely, p62 was reduced during the first 2 months but increased significantly at 4 months. Immunohistochemistry demonstrated that the proteins of LC3B and p62 were both significantly increased in the mice with diabetes at 4 months (Figure 2C). In addition, qRT-PCR disclosed an enhancement of *lc3b* and *p62* (Figure 2D). These findings suggested that autophagy might change dynamically in LECs, with an augmentation in the early HG treatment, but a blockage after consistent HG stimuli.

Compared with the LECs in normal mice, there were increased expressions of the oxidative stress markers of SOD2 and CAT in the cells subjected to hyperglycemia in the first 2 months, but an obvious reduction at 4 months (Figure 2B), which was in line with a previous study about the corneas of diabetic mice [21]. The proteins of SOD2 and CAT were both significantly reduced in the diabetic mice at 4 months as observed by immunohistochemistry (Figure 2C). qRT-PCR also showed the lower expressions of *sod*2 and *cat* (Figure 2D). Taken together, the biphasic reaction of the oxidative stress markers suggested an adaptive response during the early stage of HG treatment, followed by the failure of antioxidant biochemical defenses in the longer period of diabetes without controlling the level of blood glucose.

### Alteration of autophagy and oxidative stress in LECs upon HG stress

**Figure 3:**
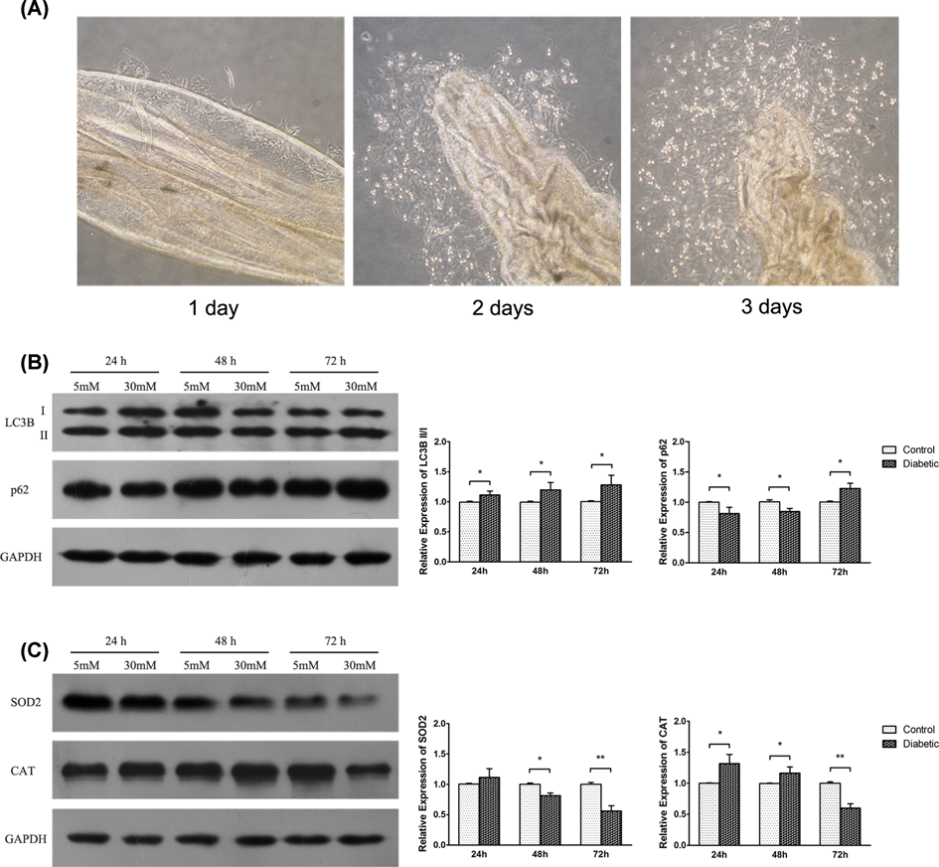
The expressions of autophagy and oxidative stress substrates in LECs exposed to normal glucose (5 mM, NG as control group) and high glucose (30 mM, HG as diabetic group) over 24, 48, and 72 h (**A**) Growing LECs obtained from the cultured mouse capsular bags. LECs attached to the bottom of the culture dish within the first day, migrated and expanded on the second day, and shrank on the third day. Western blot disclosed the markers of autophagy (**B**) and oxidative stress (**C**) in LECs exposed to NG and HG for 24, 48, and 72 h. The LC3B II/I ratio was higher at all observation time points, but p62 was down-regulated during the first 48 h and then up-regulated after 72 h in LECs exposed to high glucose; SOD2 and CAT were increased during the first 24 h and then decreased in LECs after 72 h of incubation in high glucose. Data are displayed as mean ± SD (Student’s *t*-test; B and C, *n*=3).

The LECs on the capsular bag attached to the bottom of the culture dish within the first day. On the second day, all cultured samples demonstrated anterior LEC migration and expansion from the capsular edge, which resulted in the shrinkage of the capsular bag on the third day (Figure 3A).

**Figure 4:**
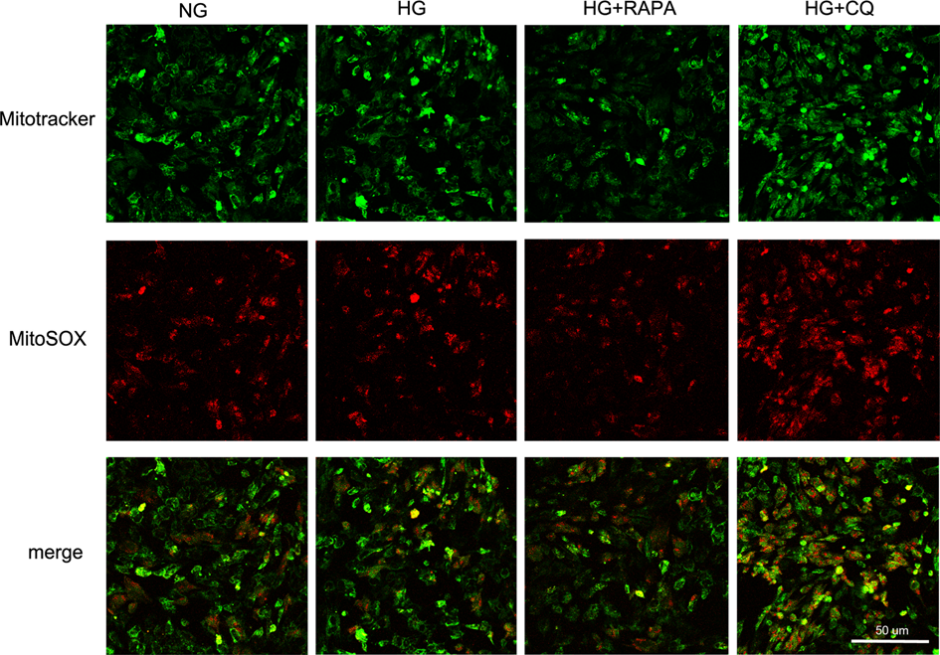
Mitochondrial ROS generation after treatment with either normal glucose (NG) or high glucose (HG) for 72 h, followed by co-treated with rapamycin (HG + RAPA) for 24 h or chloroquine (HG + CQ) for 12 h in HG conditions High glucose induced more ROS generation by LECs than the normal glucose after 72-h incubation. Rapamycin reduced ROS generation, while chloroquine enhanced the production of ROS in high glucose for 72 h, compared with the high glucose treatment alone.

The LECs were further evaluated after incubation with 5 or 30 mM glucose. Compared with the NG group, the LC3B II/I ratio was higher at all observation time points, but the expression of p62 was down-regulated during the first 48 h and up-regulated after 72 h in the HG group (Figure 3B), which was in agreement with previous studies on the human umbilical vein endothelial cells [28,29]. The levels of SOD2 and CAT were increased at 24 h and then decreased significantly in the HG-treated LECs after 72 h (Figure 3C), which was similar to the result reported by Raju et al. [20]. Moreover, more ROS generation was found in the HG group after 72-h incubation (Figure 4).

### Effects of rapamycin and chloroquine on LECs under HG conditions

**Figure 5:**
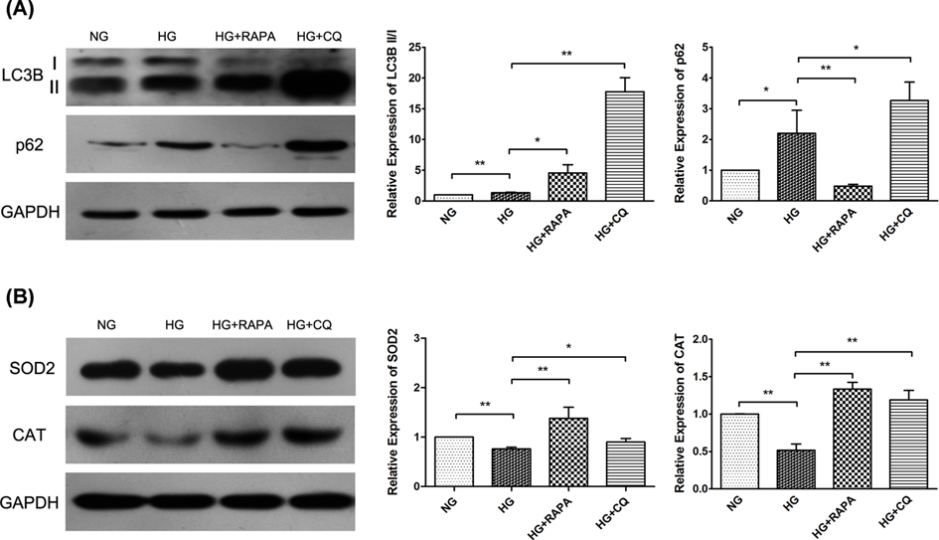
The expressions of autophagy and oxidative stress substrates in LECs after treatment with either normal glucose (NG) or high glucose (HG) for 72 h, followed by co-treated with rapamycin (HG + RAPA) for 24 h or chloroquine (HG + CQ) for 12 h in HG conditions The Western blot analysis of the markers of autophagy (**A**) and oxidative stress (**B**) in LECs. The LC3B II/I ratio was higher, and p62 was reduced in the group of HG + RAPA than in the HG alone group, with increased expressions of the two markers in the HG + CQ group. There were increased levels of oxidative stress markers in both the HG + RAPA group and the HG + CQ group compared with the HG alone group, but the difference between the groups of NG and HG+CQ was not significant. *P<0.05, **P<0.01, Student’s *t*-test (A and B, *n*=3).

The LECs were co-treated with rapamycin (HG+RAPA) as a positive control for autophagy induction and chloroquine (HG+CQ) as an inhibitor of autophagy. Rapamycin remarkably protected the LECs exposed to HG by reducing ROS generation in the group of HG + RAPA, while chloroquine enhanced the production of ROS in the HG + CQ group compared with the HG group over a period of 72 h (Figure 4). Western blot analysis revealed that the LC3B II/I ratio was much higher, and P62 was markedly reduced in the group of HG + RAPA than in the HG group (Figure 5A). The expressions of the two markers were obviously increased when the cells were treated with chloroquine. On the other hand, treatments with rapamycin and chloroquine both significantly increased the reduced levels of oxidative stress markers under HG conditions compared with HG alone, but there was no significant difference between the groups of NG and HG+CQ (Figure 5B). Collectively, the activation of cellular autophagy induced by rapamycin antagonized the oxidant damage induced by HG, and chloroquine inhibited autophagy but returned the expressions of oxidative stress markers in HG to the levels in NG.

## Discussion

In the present study, we demonstrated that the LECs continuously exposed to a high concentration of glucose could induce dysregulation of autophagy and oxidative stress, which may help to improve our knowledge of the pathogenesis of DC. We also offered novel experimental evidence that autophagy can mediate glucose-induced oxidative stress in LECs. The finding of the blocking effects of rapamycin on autophagy inhibition in and oxidant damage to LECs might provide a new clue for the pathogenesis and therapy of cataract caused mainly by HG.

Autophagy is a complicated process and is considered as a double-edged sword for cell physiology [7,30]. On one hand, it plays a crucial role in the conserved mechanism for the degradation of cellular components to maintain the cell homeostasis under nutrient stress [31]. On the other hand, excessive autophagy may induce cell death [9]. At early stage of autophagy, cytosolic microtubule-associated protein LC3B-I is converted into LC3B-II, which is bound to the autophagosome membrane [32,33]. And the LC3 II/I ratio is regarded as an indicator of early autophagy activity [34]. P62, a well-known protein, indirectly mediates the autophagosome-lysosome fusion. As p62 is reversely associated with autophagy flux [35], its accumulation as a marker for inhibition of autophagy and its decrease can be found when autophagy is activated [36–38]. Prior studies revealed that a high level of glucose induced autophagy [39,40]. In the present study, a dynamic viewing of autophagy with an augmentation of LC3B and a biphasic response of p62 protein was presented *in vivo* and *in vitro* in mice under a sustainable HG condition. Autophagy was previously disclosed to be efficiently augmented during the first 24 h under different interference approaches [41–43], which might imply a compensatory reaction activation. However, the pathway of autophagy–lysosome was inhibited beyond the capacity of compensation by elevation of p62 in HG conditions for 4 months *in vivo* and 72 h *in vitro* in the current report, which was in line with previous studies [28,29]. The results also indicated autophagy was blocked. It might be a longer period of HG stress that caused an exhausted compensatory effect in LECs. Wignes et al. [11] discovered that autophagy was inhibited by the accumulation of p62 protein in the αB-R120G mutant lenses, which eventually led to hereditary cataract formation because of a defect in protein degradation. The enhancement of p62 in an autophagy-deficient lens by the defect of Atg5 was found to result in age-related cataract [13]. The deletion of autophagy-related gene FYCO1 was also reported to be associated with congenital cataract [12]. We previously disclosed the involvement of autophagy in the formation of DC [8]. The present study further demonstrated the autophagy change from activation at the early stage to later blockage during a long period of HG stress.

Hyperglycemia induces oxidative stress and decreases the antioxidant capacity of LECs, which may contribute to the evolution of DC [44,45]. Sustained high blood glucose during the progression of diabetes leads to a redox imbalance due to the overproduction of ROS [46], which is a major cause of cellular damage [47]. SOD2 and CAT are both scavenger antioxidant enzymes. SOD2 converts superoxide anion into peroxide, which is then metabolized by CAT into water and molecular oxygen [48]. The present study speculated HG accelerated the generation of ROS in mouse LECs. In both the investigations *in vivo* and *in vitro* in mice, the expressions of oxidative stress factors showed dynamic changes under HG and time-dependent conditions. SOD2 and CAT were up-regulated at the early stage of HG treatment, which was similar to the results in podocytes [40], but the down-regulated expressions were presented in the LECs of diabetic mice for 4 months and the mouse capsular bag cultured for 72 h *in vitro*. These findings suggested an adaptive response against oxidative stress in LECs during the early stage of intervention, followed by insufficiency of antioxidant biochemical defenses that might be caused by a longer course of HG stimuli, which was consistent with previous studies [20,21]. In addition, the SOD1-null diabetic mice could much easily develop cataract than the diabetic wild-type mice [49]. The activities of SOD and CAT were lower in diabetic samples than senile populations [50]. Decreased antioxidant activity might lead to oxidation and aggregation of lens proteins, resulting in cataract [51]. Therefore, the time of HG treatment may be associated with the dysregulation of oxidative stress.

Rapamycin and chloroquine were applied to further analyze the autophagy machinery in the present study. Rapamycin is an agonist of autophagy that acts through inhibiting mTOR [52,53]. Chloroquine is a lysosomal inhibitor that reverses autophagy by accumulating autolysosomes and increasing the pH and inactive lysosomal hydrolysates. As expected, rapamycin promoted the autophagy flux, and the protective effect of rapamycin was accompanied by the LC3 II/I ratio augment and p62 degradation, while chloroquine further inhibited the autophagy pathway as evidenced by a much higher level of p62. Rapamycin was reported to rescue cardiac and skeletal muscle function in animal models of cardiomyopathy [54,55]. It was also used to prolong the life span of primates by improving the autophagy function of cells [56]. Our study presented that rapamycin prevented the autophagy inhibition in LECs treated by HG for 72 h, which might help delay the onset of cataracts in diabetic patients.

It was reported that the interplay between oxidative stress and autophagy played a critical role in the diabetes complications [57]. Some studies showed that rapamycin increased the cell viability by inducing autophagy and inhibiting oxidative stress upon HG stress [28,29]. By contrast, other researchers found that the suppression of autophagy had a protective effect in HG-induced cell injury [58,59]. The effect of autophagy could be distinct depending on the cell type and environment. Nonetheless, rapamycin could reactivate the antioxidant response program by improving the expressions of some related antioxidant enzymes [60–62], which was in agreement with our results. And growing evidences stated autophagy could fight against diabetes-induced oxidative injury [63,64]. The results of the present study indicated that rapamycin attenuated the oxidative stress by increasing the levels of oxidative stress markers and reversing the mitochondrial ROS generation in the LECs exposed to HG for 72 h of incubation. Moreover, the accumulation of chloroquine in lysosomes was discovered to increase mitochondrial ROS levels [65], which was similar to our results. Chloroquine also had a negative effect on glucose metabolism by reducing the activity of the G6PDH in the pentose phosphate pathway [65]. Therefore, the results of LECs co-treated with HG and chloroquine might parallel to the results of LECs exposed to NG. In our study, the levels of oxidative stress markers in the group of HG and chloroquine were significantly increased compared with the HG group, but had no significant difference from the NG group. The relationship of chloroquine, glucose metabolism, and oxidative stress needs to be further verified. In addition, prior studies indicated that antioxidants significantly inhibited HG-induced autophagy [41] and delayed the onset and the progression of DC [66–68]. The combination of the agonists of antioxidant and autophagy should be investigated for prevention or delay of the development of DC.

In conclusion, dynamic dysregulation of autophagy and oxidative stress in mouse LECs was caused by prolonged exposure to a high concentration of glucose. An agonist of autophagy could not only improve the level of the blocked autophagy but also prevent failure of redox-sensitive response mechanism in LECs. Complete elucidation of the mechanism of autophagy, oxdative stress, and HG in the lens epithelium will help better understand the pathogenesis of DC.

## Supplementary Material

Supplementary Figure S1 and Table S1

## Abbreviations

CAT: catalase
DC: diabetic cataract
DM: diabetic mellitus
LC3B: microtubule-associated protein 1A/1B light chain 3
LEC: lens epithelial cell
p62: sequestosome 1
ROS: reactive oxygen species
SOD2: superoxide dismutase 2
