# Supplementary material for: Autophagy attenuates high glucose-induced oxidative injury to lens epithelial cells
Source: Biosci Rep. 2020 Mar 31;40(4):BSR20193006. doi: 10.1042/BSR20193006 (PMC7109002; doi:10.1042/BSR20193006)
Supplement: Supplementary Figure S1 and Table S1 [file BSR-2019-3006_supp.pdf]

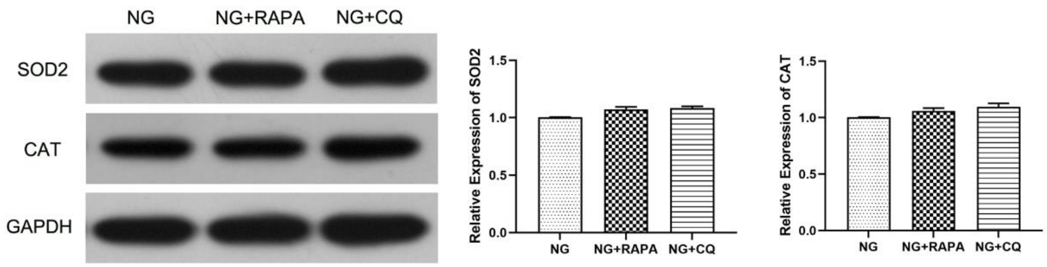


**Supplementary figure 1.** There were no difference of RAPA and CQ on SOD2 and CAT expression under non-diabetic condition.

Supplementary Table 1. The numbers of fold increase or decrease in proteins in western blots

| | LC3B II/I | P62 | SOD2 | CAT |
|---|---|---|---|---|
| 1month | 1.24 | 0.94 | 1.22 | 1.29 |
| 2 months | 1.03 | 0.81 | 1.14 | 1.17 |
| 3 months | 1.15 | 1.21 | 0.91 | 0.91 |
| 4 months | 1.27 | 1.64 | 0.60 | 0.70 |
